# MRI-based habitat radiomics and deep learning for predicting vessels encapsulating tumor clusters and survival in hepatocellular carcinoma

**DOI:** 10.1186/s13244-025-02167-3

**Published:** 2025-12-22

**Authors:** Jinjing Wang, Lixiu Cao, Hongdi Du, Yongliang Liu, Tao Zhang, Chunyan Gu, Mingzhan Du, Qian Wu, Yanfen Fan, Changhao Cao, Lingjie Wang, Yixing Yu

**Affiliations:** 1 Department of Radiology, The First Affiliated Hospital of Soochow University, Suzhou, China; 2 Department of Nuclear Medical Imaging, Tangshan People’s Hospital, Tangshan, China; 3 Department of Radiology, Suzhou Kowloon Hospital, Shanghai Jiaotong University School of Medicine, Suzhou, China; 4 Department of Neurosurgery, Tangshan People’s Hospital, Tangshan, China; 5 Department of Radiology, Nantong Third People’s Hospital, Affiliated Nantong Hospital 3 of Nantong University, Nantong, China; 6 Department of Pathology, Nantong Third People’s Hospital, Affiliated Nantong Hospital 3 of Nantong University, Nantong, China; 7 Department of Pathology, The First Affiliated Hospital of Soochow University, Suzhou, China

**Keywords:** Hepatocellular carcinoma, Magnetic resonance imaging, Gd-EOB-DTPA, Habitat radiomics, Deep learning

## Abstract

**Objective:**

The study sought to develop and validate an MRI-based deep learning radiomics (DLR) nomogram for preoperative prediction of vessels encapsulating tumor clusters (VETC) and recurrence-free survival (RFS) in hepatocellular carcinoma (HCC).

**Materials and methods:**

The dual-center study retrospectively enrolled 625 HCC patients who underwent preoperative Gd-EOB-DTPA-enhanced MRI, including training (*n* = 296), internal (*n* = 126), and external (*n* = 203) test sets. Clinical-radiologic characteristics were selected to develop a clinical-radiologic model. Habitat radiomics and deep learning (DL) features were extracted and selected to develop the habitat radiomics and DL models using the machine learning classifiers. The DLR nomogram model was ultimately constructed by integrating univariate-selected clinical-radiologic characteristics with habitat radiomics and DL scores. Both univariable and multivariable Cox regression analyses were performed to identify independent prognostic factors and develop a prognostic model for RFS.

**Results:**

In the external test set, the DLR nomogram model yielded a higher area under the curve (AUC) than the clinical-radiologic model (0.752 vs 0.678; *p* = 0.004), while habitat radiomics (0.750) and DL models (0.748) showed comparable performance (both *p* > 0.05). The DLR nomogram consistently demonstrated the higher F1-scores across all three sets. The prognostic model incorporating AFP (hazard ratio (HR), 1.628 [95% CI: 1.113–2.380]; *p* = 0.012) and DLR score (1.279 [1.051–1.557]; *p* = 0.014) achieved C-indexes of 0.679 and 0.642 for RFS in the internal and external test sets.

**Conclusion:**

The DLR nomogram model helps predict VETC in HCC and assess the risk for RFS.

**Critical relevance statement:**

Interpretable deep learning radiomics nomogram model provides clinicians with more precise technical support for preoperative prediction of VETC status and RFS in HCC, potentially aiding in clinical decision-making and follow-up strategies.

**Key Points:**

- Vessels encapsulating tumor clusters (VETC) is a critical predictor of aggressive hepatocellular carcinoma.
- The deep learning radiomics (DLR) nomogram model helps predict VETC, and the DLR score serves as an independent prognostic factor for recurrence-free survival.
- The model demonstrated favorable interpretability through the SHAP method.

**Graphical Abstract:**

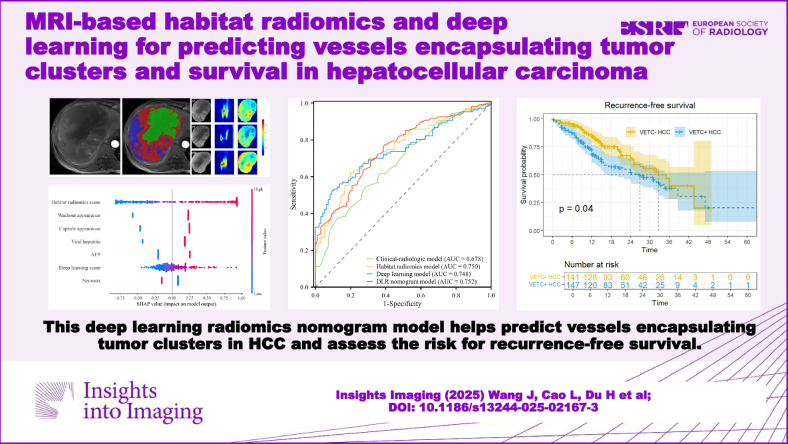

## Introduction

Hepatocellular carcinoma (HCC) is the third leading cause of cancer-related death and has a poor prognosis with postoperative 5-year recurrence rates of 50–70%, which is a major health problem worldwide [1, 2]. Angiogenesis and vascular remodeling of HCC are closely associated with tumor progression [3–5]. The vessels encapsulating tumor clusters (VETC) is a novel and distinct aggressive vascular pattern in HCC. It is defined as the presence of CD34+ sinusoidal vessels encapsulating tumor clusters in pathological imaging [6], and is known to promote hematogenous metastasis and early recurrence [7–9]. Studies have shown that patients with VETC+ HCC can benefit from sorafenib or postoperative prophylactic adjuvant transarterial chemoembolization (TACE), with improvements in both overall survival and recurrence-free survival (RFS) [10, 11], whereas similar benefits are not observed in VETC− patients. Thus, accurate preoperative prediction of VETC status has important clinical implications. Furthermore, as patients with VETC + HCC face a higher risk of recurrence, preoperative prediction of RFS may help guide individualized surveillance strategies.

However, VETC can currently only be diagnosed by postoperative histopathology. Therefore, it is necessary to develop a non-invasive tool to preoperatively predict VETC status and RFS.

Recent studies have explored the preoperative prediction of VETC status and patient prognosis through medical image analysis, particularly radiomics and deep learning. Nevertheless, conventional radiomics typically treats the entire tumor as a homogeneous entity [12]. Habitat radiomics is an innovative radiomics strategy that employs voxel-level radiomics clustering algorithms to delineate biologically homogeneous subregions within tumors for feature extraction [13, 14].

Deep learning (DL) has played a crucial role in disease diagnosis; however, its clinical utility is limited by an inherent lack of interpretability [15–17]. SHAP (Shapley additive explanation) is a tool to achieve model interpretability, which provides an interpretable framework based on cooperative game theory and can quantify the contributions of features in models globally and individually [18, 19]. Furthermore, the integration of multimodal data, such as habitat radiomics and DL features, can offer more comprehensive information, which provides better predictive and prognostic performance [20–22].

Therefore, we attempted to develop and validate a deep learning radiomics (DLR) nomogram model by integrating selected clinical-radiologic characteristics with habitat radiomics and DL scores based on Gd-EOB-DTPA-enhanced MRI to preoperatively predict VTEC status and RFS in HCC.

## Materials and methods

### Patient population and clinical characteristics

**Fig. 1:**
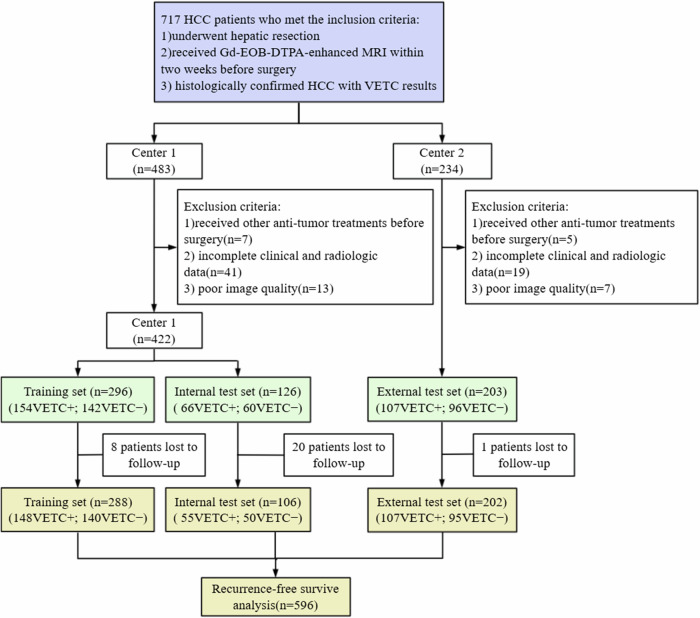
The process of patient selection. HCC, hepatocellular carcinoma; VETC, vessels encapsulating tumor clusters

This dual-center retrospective study was approved by the institutional ethics review board of the First Affiliated Hospital of Soochow University (Approval No. 2024-260), with informed consent waived. From January 2016 and March 2024, 717 patients with HCC were consecutively enrolled. The inclusion criteria were: (1) underwent hepatic resection; (2) received Gd-EOB-DTPA-enhanced MRI within 2 weeks before surgery; (3) histologically confirmed HCC with VETC results. The exclusion criteria were: (1) received other anti-tumor treatments before surgery; (2) incomplete clinical-radiologic data; (3) poor image quality. The largest lesion was selected for analysis when multiple lesions were present. Ultimately, 625 eligible patients were included for subsequent analysis. Patients from the First Affiliated Hospital of Soochow University (center 1) were randomly divided to the training (*n* = 296) and internal test sets (*n* = 126) at a ratio of 7:3, while patients from Affiliated Nantong Hospital 3 of Nantong University (center 2) were assign to the external test set (*n* = 203) (Fig. 1).

**Fig. 2:**
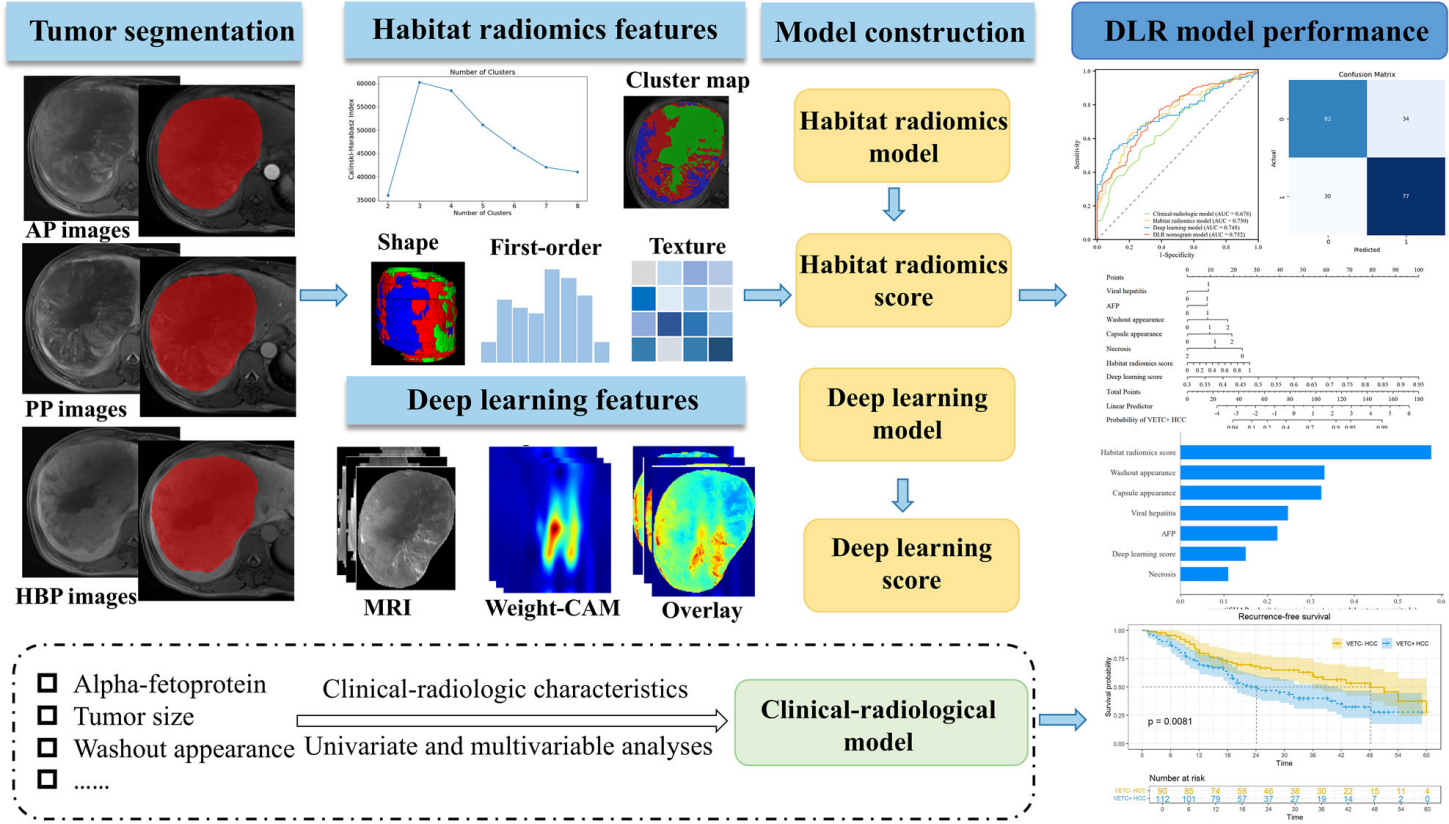
The overall workflow of this study. The process from tumor segmentation to model construction for predicting vessels encapsulating tumor clusters and recurrence-free survival in hepatocellular carcinoma. DLR, deep learning radiomics; AP, arterial phase; PP, portal venous phase; HBP, hepatobiliary phase

Clinical characteristics, including age, sex, viral hepatitis, alanine aminotransferase (ALT), aspartate aminotransferase (AST), γ-glutamyl transferase (GGT), alpha-fetoprotein (AFP), and cirrhosis, were collected from the medical records. The overall workflow of this study is illustrated in Fig. 2.

The CLEAR checklist was applied to ensure standardized reporting, and the completed checklist is available as supplementary material.

### Pathological analysis

**Fig. 3:**
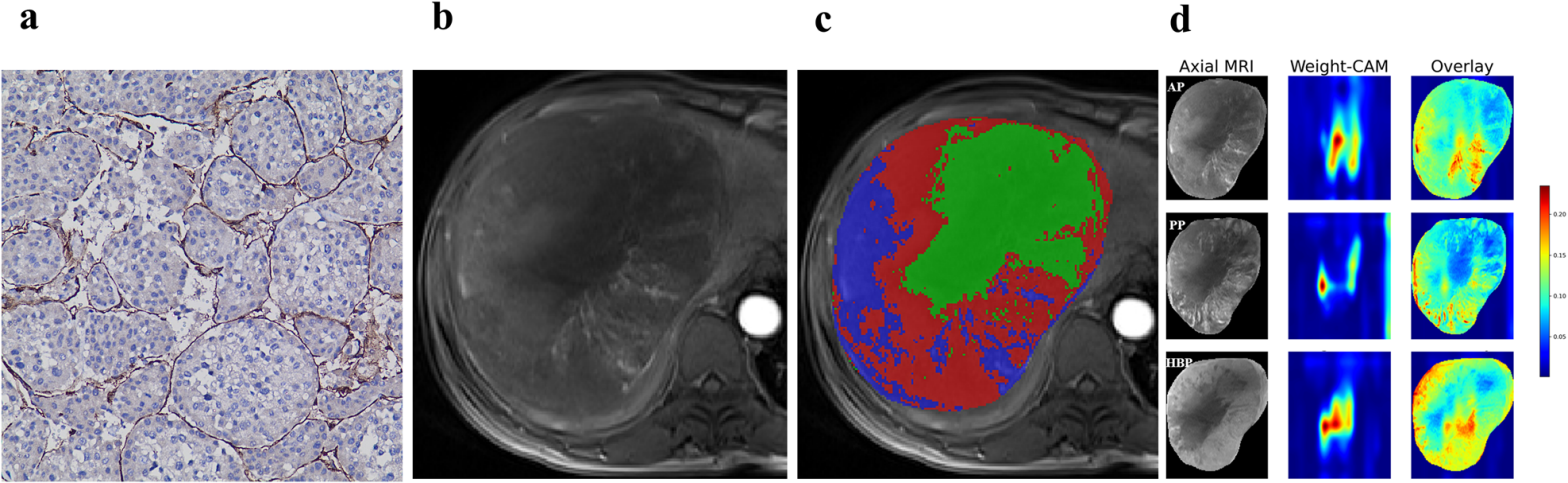
Digital pathology image and models visualization based on a 44-year-old male patient with VETC + HCC. **a** Immunohistochemical staining of CD34 (original magnification, × 200) in a VETC+ patient. **b**, **c** Habitat radiomics model visualization. Demonstration of three subregions clustered by similar voxels using AP images. The HCC lesion showing predominantly mild-to-moderate enhancement (red habitats) and necrotic habitats (green habitats), with only a few hypervascular habitats (blue habitats). **d** Deep learning model visualization. The class activation mapping (CAM) heatmaps of the axial AP, PP, and HBP images. Red signifying the activation region associated with the VETC pattern. VETC, vessels encapsulating tumor clusters; HCC, hepatocellular carcinoma; AP, arterial phase; PP, portal venous phase; HBP, hepatobiliary phase

All the tumor specimens were evaluated by two pathologists (M.D. and C.G., each with 8 years of experience in hepatic histopathology). If there was a disagreement, a senior pathologist with more than 20 years of experience would be consulted. The VETC pattern was characterized by the presence of sinusoid-like vessels encapsulating tumor clusters with CD34+ endothelium (Fig. 3a). Cases with a visible VETC pattern, either entirely or partially, were identified as VETC+, while those without any VETC pattern were identified as VETC−.

### MRI acquisition and tumor segmentation

Two 3.0 T MRI scanners (Siemens Magnetom Skyra and GE SIGNA Premier) were used to perform the scans. After a bolus injection of 0.1 mL/kg body weight of Gd-EOB-DTPA (Primovist®, Bayer), multiphase images including arterial phase (AP), portal venous phase (PP), and hepatobiliary phase (HBP) images were obtained at 30–35 s, 65–70 s, and 20 min, respectively. The MRI scan sequences and parameters are shown in Supplementary Table S1.

Volumes of interest (VOIs) of HCCs in three phases were manually delineated in consensus by two junior radiologists (Q.W. and Y.F., with 5 and 8 years of experience) using ITK-SNAP (http://www.itksnap.org) and reviewed by a senior radiologist with 12 years of experience (Y.Y.). Inter-observer reproducibility was assessed using the average Dice similarity coefficient (DSC) based on all segmentation masks to assess the reproducibility between the segmentations from two junior radiologists and the senior radiologist.

### Clinical-radiologic model construction

Two abdominal radiologists (Q.W. and Y.F.) assessed the following radiologic characteristics in consensus: ①tumor size; ②tumor number; ③lesion shape; ④enhancement patterns: rim arterial phase hyperenhancement (APHE), non-rim APHE, hypo-enhancement; ⑤washout appearance: peripheral washout, non-peripheral washout, absent; ⑥capsule appearance: enhancing capsule, non-enhancing capsule, absent; ⑦necrosis: focal necrosis, substantial necrosis (occupying at least 20% of the tumor at the largest cross-sectional diameter) [23, 24], none; ⑧intratumor artery; ⑨delayed enhancement; ⑩satellite nodules; ⑪intratumor hemorrhage; ⑫intratumor fat; ⑬LI-RADS. All included patients met the LI-RADS v2018 high-risk population criteria and were therefore eligible for LI-RADS categorization [25]. If any discrepancies arose, the final assessment was adjudicated by a senior radiologist (Y.Y.). Representative images of key qualitative features are shown in Supplementary Fig. S1. Based on the training set, features selected by univariate analysis were tested for multicollinearity by excluding variables with a variance inflation factor (VIF) exceeding 10. The remaining features were incorporated into the multivariable logistic regression to identify independent predictors and develop clinical-radiologic models for VETC prediction.

### Habitat radiomics model construction

VOIs were imported into the PyRadiomics package (v3.1, http://www.radiomics.io/pyradiomics.html), which conforms to the Image Biomarker Standardization Initiative (IBSI) standards [26], for habitat radiomics features extraction. Image preprocessing included image normalization, isotropic resampling to 1 × 1 × 1 mm³, discretization with a fixed bin width of 5, and wavelet and LoG filters. K-means clustering (k = 2–8) with the Calinski-Harabasz index identified optimal k = 3 for subregion segmentation and heterogeneity visualization (Fig. S2). Pixels from the same cluster were assigned the same color to generate a clustering label map (Fig. 3b, c). A total of (*n* = 1132 × k) features were extracted from each patient.

Features from all three sets were imported into FAE software (v0.5.6, https://github.com/salan668/FAE) for processing, an open-source software package programmed with Python. Feature preprocessing included data balancing with synthetic minority over-sampling technique, Z-score normalization, and removal of highly correlated features (PCC > 0.90). Then, within the FAE framework, four feature selection methods (ANOVA, Kruskal-Wallis, RFE, and Relief) were combined with 9 classifiers (SVM, LDA, Random Forest, Logistic Regression, LR-Lasso, AdaBoost, Decision Tree, Gaussian Process, Naïve Bayes) using default scikit-learn (v1.3.0) hyperparameters, yielding 144 (4 × 9 × 4) models for AP, PP, HBP, and combined phase (CP). All models were trained on a fixed training set, and no cross-validation was performed. The optimal habitat radiomics model for VETC prediction was selected based on the highest area under the curve (AUC), and habitat radiomics scores were derived from its mean prediction probability. Finally, the habitat radiomics model based on the RFE method and the SVM classifier was identified as the optimal model.

### Deep learning model construction

Our study developed 3D Swin Transformer models to extract features from intensity-normalized ([0,1]) MRI images. The models utilized the “swin3d_s” architecture from the ‘torchvision.models.video’ module in PyTorch (v2.1.0, https://github.com/pytorch/pytorch). Data augmentation (Resized, ScaleIntensityRanged, RandFlipd, RandAffined), learning rate decay (initial 0.0001, exponential decay 0.1/7 epochs), and focal loss (γ = 2.0, α = 0.3) were incorporated into training to address overfitting and class imbalance. Training used a batch size of 16 for 200–500 epochs. The final weights were used to extract features from the images. Finally, 768 DL features were extracted for each patient. These features were processed in FAE software following the same modeling pipeline as the habitat radiomics model, which combined four feature selection methods with 9 classifiers using default scikit-learn hyperparameters, resulting in 144 DL models. All models were trained on a fixed training set without cross-validation. The optimal DL model was selected by the highest AUC, and DL scores were calculated by its mean predicted probabilities. Finally, the DL model based on the Kruskal–Wallis test and Naïve Bayes classifier was identified as the optimal model. Class activation mapping (CAM) highlighted regions contributing to predictions [27] (Fig. 3d).

### Deep learning radiomics nomogram model construction and visualization

The DLR nomogram model for VETC prediction was developed by integrating univariate-selected clinical-radiologic characteristics (from the training set), along with habitat radiomics and DL scores, using multivariable logistic regression after assessing multicollinearity. The DLR score was calculated from the mean predicted probability. SHAP analysis provided intuitive visual interpretations of the model.

### Cox regression analysis for RFS

Postoperative follow-up was conducted every 3–6 months routinely through AFP and radiological examinations (enhanced CT or MRI) to identify the disease recurrence or metastasis. RFS was defined as the time from surgery to the first documented tumor recurrence or death from any cause before January 26, 2025.

Based on the training set, univariable Cox regression analysis was performed to identify potential prognostic factors of RFS among the DLR score and ten clinical-radiologic characteristics (age, sex, viral hepatitis status, ALT, AST, GGT, AFP level, cirrhosis, tumor size, and tumor number). Correlation analysis and multicollinearity assessment were used to exclude redundant variables. Multivariable Cox regression analysis was performed to identify independent predictors and develop a prognostic model for RFS.

### Statistical analysis

All statistical analyses were performed with SPSS 25.0, R software (v4.4.1, https://www.r-project.org), and Python (v3.8.8, https://www.python.org). Quantitative variables were recorded as mean ± standard deviation or median with interquartile range and compared by independent *t-*test or Mann–Whitney *U-*test. Categorical variables were recorded as frequencies with percentages and compared by χ²-test or Fisher test. Model diagnostic performance was evaluated using AUCs, compared via Delong test, with optimal cutoff selected by maximizing the Youden index. The receiver operating characteristic (ROC) curves and nomogram were plotted using “pROC” and “rms” R package. Calibration was assessed using calibration curves and the Hosmer-Lemeshow goodness-of-fit test with “rms” and “ResourceSelection” R package, respectively. SHAP analysis was conducted using Python with “shap” and “scikit-learn” packages. Survival analysis was conducted using Kaplan–Meier analysis (“survival” and “survminer” R packages), and prognostic performance was assessed with the C-index (“survival” package). A two-tailed *p* < 0.05 was considered statistically significant.

## Results

### Clinical-radiologic characteristics of patients

A total of 625 patients (mean age 59.32 ± 10.31 years; 473 men) from two centers were included. Univariate analysis identified two clinical and ten radiologic characteristics significantly associated with VETC status, including viral hepatitis, AFP, tumor size, enhancement patterns, washout appearance, capsule appearance, necrosis, intratumor artery, delayed enhancement, satellite nodules, intratumor hemorrhage, and LI-RADS (*p* < 0.05; Tables 1, 2). No multicollinearity was observed (VIF 1.02–1.35).

**Table 1:** Clinical characteristics of patients in three sets

Variables	Training set (*n* = 296)				Internal test set (*n* = 126)	External test set (*n* = 203)	*p*-value*	*p*-value^#^
	Total (*n* = 296)	VETC+ (*n* = 154)	VETC− (*n* = 142)	*p*-value				
Age	60.11 ± 10.87	59.84 ± 10.44	60.41 ± 11.34	0.66	59.17 ± 10.93	58.24 ± 8.93	0.42	**0.04**
Sex				0.07			0.77	**0.02**
Male	234 (79.1)	128 (83.1)	106 (74.6)		98 (77.8)	141 (69.5)		
Female	62 (20.9)	26 (16.9)	36 (25.4)		28 (22.2)	62 (30.5)		
Viral hepatitis	217 (73.3)	121 (78.6)	96 (67.7)	**0.03**	93 (73.8)	185 (91.1)	0.92	**< 0.001**
ALT > 50 U/L	67 (22.6)	39 (25.3)	28 (19.7)	0.25	16 (12.7)	48 (23.6)	**0.02**	0.79
AST > 40 U/L	81 (27.4)	49 (42.1)	32 (22.5)	0.07	38 (30.2)	215 (72.6)	0.56	**0.002**
GGT > 60 U/L	126 (42.6)	70 (45.5)	56 (39.4)	0.30	52 (41.3)	79 (38.9)	0.81	0.42
AFP > 100 ng/mL	120 (40.5)	71 (46.1)	49 (34.5)	**0.04**	43 (34.1)	52 (25.6)	0.22	**0.001**
Cirrhosis	137 (46.3)	77 (50.0)	60 (42.3)	0.18	62 (49.2)	133 (65.6)	0.58	**< 0.001**

Bold font indicates a significant difference

*p*-values comparing VETC + HCC with VETC − HCC in the training set; *p*-values* comparing the training set with internal test set; *p*-values^#^ comparing the training set with external test set

*ALT* alanine aminotransferase, *AST* aspartate aminotransferase, *GGT* γ-glutamyl transferase, *AFP* alpha-fetoprotein

**Table 2:** Radiologic characteristics of patients in three sets

Variables	Training set (*n* = 296)				Internal test set (*n* = 126)	External test set (*n* = 203)	*p*-value*	*p*-value^#^
	Total (*n* = 296)	VETC+ (*n* = 154)	VETC− (*n* = 142)	*p*-value				
Tumor size	4.15 (2.83, 7.18)	5.41 (3.40, 5.15)	3.35 (2.40, 5.20)	**< 0.001**	4.55 (2.48, 7.50)	2.50 (1.70, 4.20)	0.87	**< 0.001**
Tumor number				0.09			0.91	**0.001**
1	205 (69.3)	100 (64.9)	105 (98.3)		88 (69.8)	167 (82.3)		
≥ 2	91 (30.7)	54 (35.1)	37 (26.1)		38 (30.2)	36 (17.7)		
Lesion shape				0.08			0.32	0.89
Round	198 (66.9)	96 (62.3)	102 (71.8)		78 (61.9)	137 (67.5)		
Lobulated	98 (33.1)	58 (37.7)	40 (28.2)		48 (38.1)	66 (32.5)		
Enhancement patterns				**0.03**			0.74	0.48
Rim APHE	29 (9.8)	13 (8.4)	16 (11.3)		10 (7.9)	22 (10.8)		
Non-rim APHE	250 (84.5)	137 (89.0)	113 (79.6)		107 (84.9)	165 (81.3)		
Hypo-enhancement	17 (5.7)	4 (2.6)	13 (9.2)		9 (7.1)	16 (7.9)		
Washout appearance				**0.001**			0.36	**0.004**
Peripheral	17 (5.7)	7 (4.5)	10 (7.0)		5 (4.0)	15 (7.4)		
Non-peripheral	191 (64.5)	115 (74.7)	76 (53.5)		90 (71.4)	154 (75.9)		
Absent	88 (29.7)	32 (20.8)	56 (39.4)		31 (24.6)	34 (16.7)		
Capsule appearance				**0.001**			0.25	**< 0.001**
Enhancing	149 (50.3)	90 (58.4)	59 (41.5)		57 (45.2)	101 (49.8)		
Non-enhancing	40 (13.5)	24 (15.6)	24 (15.6)		13 (10.3)	7 (3.4)		
Absent	107 (36.1)	40 (26.0)	67 (47.2)		56 (44.4)	95 (46.8)		
Necrosis				**0.004**			0.77	**0.02**
Focal	92 (31.1)	61 (39.6)	31 (21.8)		39 (31.0)	40 (19.7)		
Substantial	22 (7.4)	9 (5.8)	13 (9.2)		12 (9.5)	15 (7.4)		
None	182 (61.5)	84 (54.5)	98 (69.0)		75 (59.5)	148 (72.9)		
Intratumor artery	118 (39.9)	80 (51.9)	38 (26.8)	**< 0.001**	56 (44.4)	52 (25.6)	0.38	**0.001**
Delayed enhancement	84 (28.3)	31 (20.1)	53 (37.3)	**0.001**	31 (24.6)	38 (18.7)	0.63	**0.02**
Satellite nodules	50 (16.9)	35 (22.7)	15 (10.6)	**0.005**	19 (16.4)	7 (3.4)	0.65	**< 0.001**
Intratumor hemorrhage	59 (19.9)	39 (25.3)	20 (14.1)	**0.02**	34 (27.0)	25 (12.3)	0.11	**0.03**
Intratumor fat	67 (22.6)	29 (18.8)	38 (26.8)	0.10	25 (19.8)	46 (22.7)	0.53	0.99
LI-RADS				**< 0.001**			0.46	**0.003**
LR-3	16 (5.4)	3 (1.9)	13 (9.2)		11 (8.7)	24 (11.8)		
LR-4	50 (16.9)	18 (11.7)	32 (22.5)		17 (13.5)	20 (9.9)		
LR-5	198 (66.9)	121 (78.6)	77 (54.2)		87 (69.0)	147 (72.4)		
LR-M	32 (10.8)	12 (7.8)	20 (14.4)		11 (8.7)	12 (5.9)		

Bold font indicates a significant difference

*p*-value comparing VETC + HCC with VETC − HCC in the training set; *p*-values* comparing the training set with internal test set; *p*-values^#^ comparing the training set with external test set

*APHE* arterial phase hyperenhancement, *No APHE* hypo-enhancement without arterial phase hypervascularity

### Performance of clinical-radiologic model

Four independent predictors of the clinical-radiologic model were obtained using multivariable logistic regression analysis, including tumor size, viral hepatitis, absence of washout, and focal/substantial necrosis (Table 3). The model yielded AUCs of 0.696 (95% CI: 0.608–0.775) in the internal test set and 0.678 (95% CI: 0.609–0.742) in the external test set (Table 4).

**Table 3:** Multivariable logistic regression analyses of two models in the training set

Variables	Clinical-radiologic model				Deep learning radiomics nomogram model		
	β	OR (95% CI)	*p*-value		β	OR (95% CI)	*p*-value
Tumor size	0.25	1.28 (1.15–1.42)	**< 0.001**				
Viral hepatitis	−0.80	0.45 (0.25–0.82)	**0.01**		−0.68	0.50 (0.26–0.97)	**0.04**
Washout appearance							
Peripheral	−0.07	0.93 (0.27–3.24)	0.91		−0.01	0.98 (0.25–3.91)	0.99
Non-peripheral	0.83	1.28 (1.15–1.42)	0.18		1.05	2.84 (0.73–11.00)	0.13
Absent	0	1	**0.01**		0	1	**0.004**
Capsule appearance							
Enhancing	−0.73	0.48 (0.21–1.11)	0.08		−0.99	0.37 (0.15–0.92)	0.33
Non-enhancing	−0.11	0.90 (0.40–2.00)	0.79		−0.28	0.75 (0.32–1.79)	0.52
Absent	0	1	0.08		0	1	**0.04**
Necrosis							
Focal	1.19	3.29 (1.01–10.74)	**0.05**		1.90	6.68 (1.80–24.83)	**0.01**
Substantial	1.22	3.37 (1.07–10.65)	**0.04**		1.49	4.42 (1.23–15.89)	**0.02**
None	0	1	0.11		0	1	**0.02**
AFP					−0.66	0.52 (0.29–0.94)	**0.03**
Habitat radiomics score					0.66	1.93 (1.37–2.72)	**< 0.001**
Deep learning score					1.03	2.80 (1.83–4.30)	**< 0.001**

Bold font indicates a significant difference

*OR* odds ratio, *CI* confidence interval, *AFP* alpha-fetoprotein, *β* coefficients calculated from the multivariable logistic regression model

**Table 4:** Performance of four predictive models in three sets

Models and sets	AUC (95% CI)	F1-score	Accuracy	Sensitivity	Specificity	Precision	Recall
Clinical-radiologic model							
Training set	0.761 (0.661–0.767)	0.680 (0.656, 0.764)	66.55%	64.29%	74.65%	67.74%	68.18%
Internal test set	0.696 (0.608–0.775)	0.657 (0.522, 0.725)	62.70%	40.91%	91.67%	63.38%	68.18%
External test set	0.678 (0.609–0.742)	0.573 (0.533, 0.693)	61.08%	54.21%	71.87%	67.95%	49.53%
Habitat radiomics model							
Training set	0.716 (0.661–0.767)	0.638 (0.612, 0.752)	65.54%	68.83%	69.01%	70.31%	58.44%
Internal test set	0.749 (0.664–0.822)	0.667 (0.538, 0.732)	69.05%	57.58%	85.00%	76.47%	59.09%
External test set	0.750 (0.684–0.808)	0.361 (0.340, 0.533)	58.13%	61.68%	80.21%	92.31%	22.43%
Deep learning model							
Training set	0.711 (0.653–0.769)	0.595 (0.525, 0.662)	66.22%	43.51%	90.85%	72.22%	50.65%
Internal test set	0.709 (0.618–0.801)	0.626 (0.510, 0.716)	67.46%	48.48%	88.33%	73.47%	54.55%
External test set	0.748 (0.681–0.815)	0.704 (0.649, 0.764)	70.44%	52.34%	90.62%	56.49%	93.46%
Deep learning radiomics nomogram model							
Training set	0.822 (0.774–0.864)	0.756 (0.727, 0.825)	74.66%	83.12%	69.01%	75.82%	75.32%
Internal test set	0.715 (0.627–0.792)	0.672 (0.631, 0.715)	65.87%	46.97%	90.00%	67.69%	66.67%
External test set	0.752 (0.686–0.809)	0.706 (0.656, 0.782)	68.47%	76.64%	61.46%	69.37%	71.96%

*AUC* area under the curve, *CI* confidence interval, *Habitat radiomics model* the optimal habitat radiomics model among all phases, *Deep learning model* the optimal deep learning model among all phases

### Performance of habitat radiomics and deep learning models

The performance of the best-performing habitat radiomics and DL models in each phase is presented in Table S2. Five habitat radiomics features from HBP and 18 DL features from CP were selected to construct the optimal habitat radiomics and DL models (Table S3). These features showed low inter-feature correlation (Fig. S3). The optimal habitat radiomics model showed AUCs of 0.749 (95% CI: 0.664–0.822) and 0.750 (95% CI: 0.684–0.808), while the optimal DL model achieved AUCs of 0.709 (95% CI: 0.618–0.801) and 0.748 (95% CI: 0.681–0.815) in the internal and external test sets, respectively (Table 4). The average DSC of all masks was 0.92, indicating excellent spatial overlap. CAM heatmaps showed that the DL model exhibited significant attention toward hypervascular tumor regions (Fig. 3d).

### Deep learning radiomics nomogram model construction and visualization

**Fig. 4:**
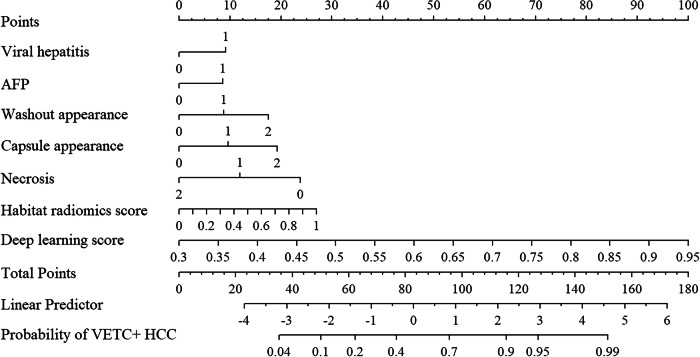
The deep learning radiomics nomogram. AFP, alpha-fetoprotein

**Fig. 5:**
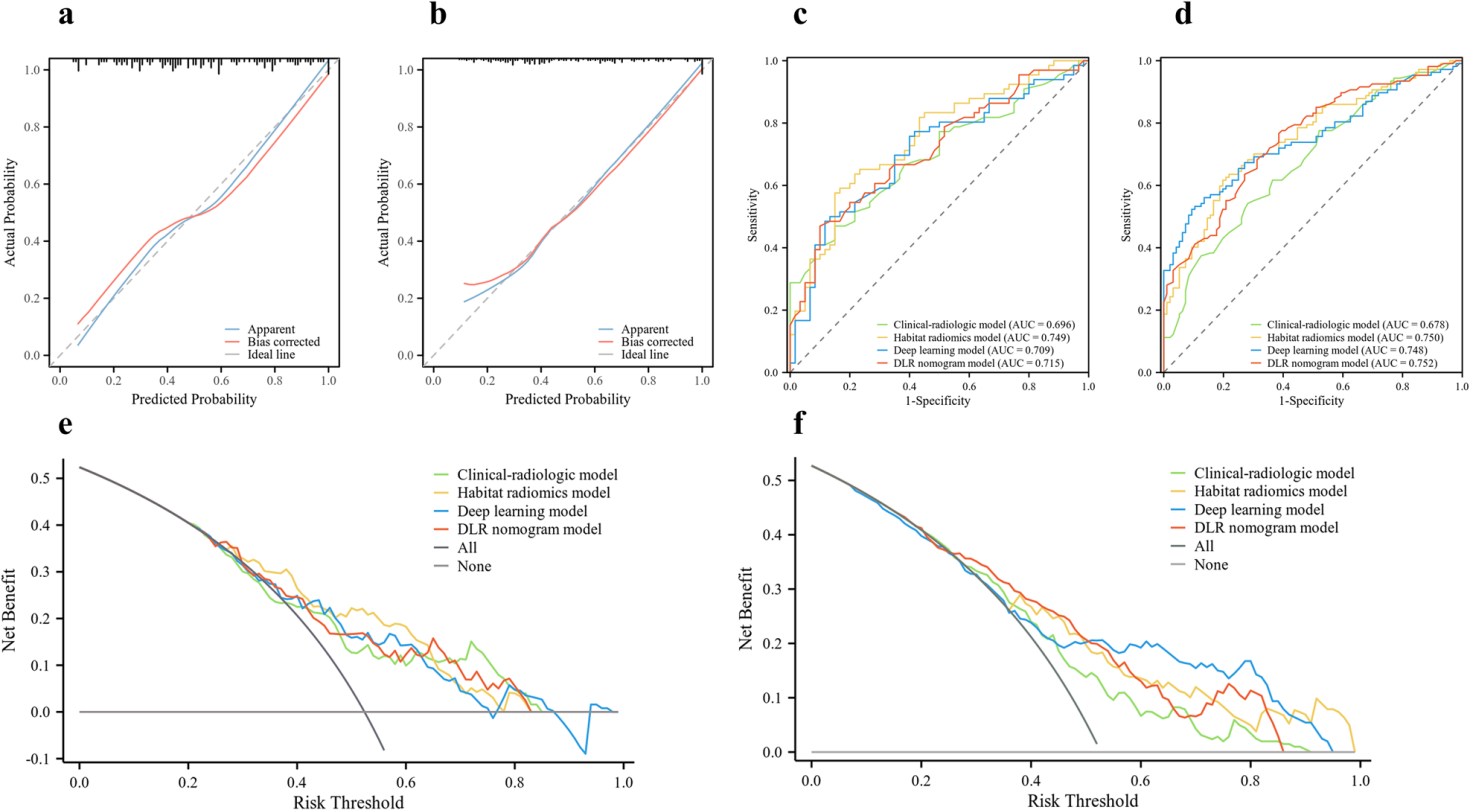
Calibration curves of the DLR nomogram model in the internal (**a**) and external test sets (**b**). ROC curves of predictive models in the internal (**c**) and external test sets (**d**). The decision curves of the predictive models in the internal (**e**) and external test sets (**f**). DLR, deep learning radiomics; ROC, receiver operating characteristic

Seven independent predictors of the DLR nomogram model were as follows: viral hepatitis, absence of washout, absence of capsule, necrosis, AFP, habitat radiomics score, and DL score (Table 3). No multicollinearity was detected with VIFs ranging from 1.06 to 2.10. The model achieved promising performance in the internal (AUC: 0.715; 95% CI: 0.627–0.792) and external test sets (AUC: 0.752; 95% CI: 0.686–0.809) and exhibited relatively high F1-scores across all three sets (Table 4) (Fig. 4). Calibration curves showed good agreement between predicted and observations, with Hosmer-Lemeshow *p*-values of 0.739 and 0.489 for the internal and external test sets (Fig. 5a, b). The detailed feature selection steps for each model are provided in Supplementary Table S4.

**Fig. 6:**
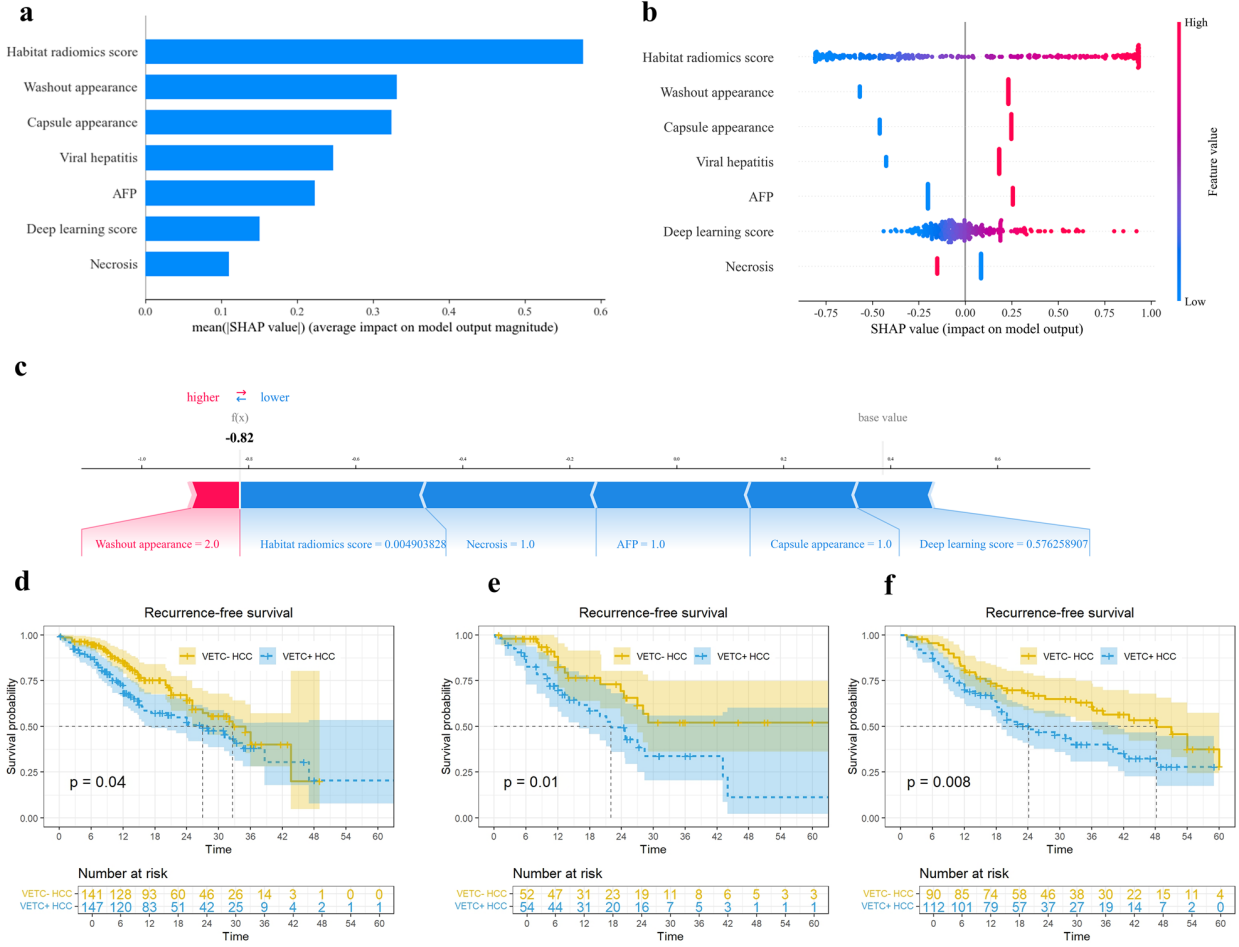
The feature importance bar-plot (**a**) and chart (**b**) ranking the 7 features of the deep learning radiomics nomogram model in the external test set based on the mean (|SHAP value|). Red color in the feature importance chart represents a higher value. The SHAP force plot (**c**) showing the predictive process of a patient with histological VETC − HCC. Blue color indicates that the feature has a negative effect on the prediction (arrow to the left, SHAP value decreases), while red color indicates that the feature has a positive effect on the prediction (arrow to the right, SHAP value increases). The length of the color bar indicates the strength of the contribution. Base value indicates the SHAP reference value, which is the mean predicted by the model based on the training set. f(x) represents the SHAP value of the individual patient. Recurrence-free survival curves of the training (**d**), internal (**e**), and external (**f**) test sets according to nomogram-predicted VETC status via Kaplan–Meier analysis. SHAP, SHapley Additive explanation; VETC, vessels encapsulating tumor clusters; HCC, hepatocellular carcinoma

Moreover, SHAP analysis revealed the habitat radiomics score and washout appearance as the top contributors to predictions (Fig. 6a, b). The SHAP force plot illustrates the predictive process for an individual patient with histological VETC − HCC (Fig. 6c).

### Model performance comparison

The DLR nomogram model significantly outperformed the clinical-radiologic model (AUC: 0.752 vs 0.678; *p* = 0.004) in the external test set and showed comparable performance in the internal test set (AUC: 0.715 vs 0.696; *p* = 0.55) (Table 5) (Fig. 5c, d). In the internal and external test sets, the DLR nomogram model (AUC: 0.715, 0.752) showed comparable AUCs compared to habitat radiomics (AUC: 0.749, 0.750; *p* = 0.40, 0.95) and DL models (AUC: 0.709, 0.748; *p* = 0.91, 0.86) (Table 5). However, it achieved higher F1-scores (F1-score: 0.672, 0.706) than habitat radiomics (F1-score: 0.667, 0.361) and DL models (F1-score: 0.626, 0.704) in the internal and external test sets (Table 5). Decision curve analysis showed the DLR nomogram provided net benefit across a broad range of threshold probabilities. (Fig. 5e, f).

**Table 5:** Performance comparisons between predictive models

Training set			Internal test set			External test set		
AUC (95% CI)	Z statistic	*p*-value	AUC (95% CI)	Z statistic	*p*-value	AUC (95% CI)	Z statistic	*p*-value
DLR nomogram model vs Clinical-radiologic model								
0.822 vs 0.761 (0.024–0.099)	3.224	0.001	0.715 vs 0.696 (−0.042 to 0.079)	0.602	0.55	0.752 vs 0.678 (0.023–0.124)	2.871	**0.004**
DLR nomogram model vs Habitat radiomics model								
0.822 vs 0.716 (0.055–0.158)	4.070	< 0.001	0.715 vs 0.749 (−0.045 to 0.113)	0.845	0.40	0.752 vs 0.750 (−0.058 to 0.063)	0.069	0.95
DLR nomogram model vs Deep learning model								
0.822 vs 0.711 (0.062–0.161)	4.386	< 0.001	0.715 vs 0.709 (−0.080 to 0.091)	0.119	0.91	0.752 vs 0.748 (−0.037 to 0.045)	0.181	0.86

Bold font indicates a significant difference

*AUC* area under the curve, *CI* confidence interval, *DLR* deep learning radiomics

### Cox regression analysis for RFS

By January 26, 2025, 596 (95%) patients were included in the RFS follow-up. The remaining 29 patients (5%) were lost to follow-up at random from the very first scheduled follow-up and were excluded from the survival cohort. Patients with VETC + HCC had poorer RFS than those with VETC − HCC in the three sets (all *p* < 0.05). The median RFS was 27.0 (95% CI: 17.3, not evaluable (NE)), 22.0 (95% CI: 16.0, NE), and 24.1 (95% CI: 19.0, 41.0) months for patients with nomogram-pred VETC + HCC and 32.6 (95% CI: 25.0, NE), NE (95% CI: 27.0, NE), and 48.2 (95% CI: 36.2, NE) months for patients with nomogram-pred VETC − HCC in the training (*p* = 0.04), internal (*p* = 0.01), and external (*p* = 0.008) test sets, respectively (Fig. 6d–f).

The clinical-radiologic characteristics and prognosis did not differ between the training and internal test sets (Table S5). For the training set, univariable Cox regression analysis showed that AST, GGT, AFP, tumor size, tumor number, and DLR score were associated with RFS (Table S6). Given the moderate correlation between the DLR score and tumor size (ρ = 0.60), and the likelihood that the DLR score captures prognostic information conveyed by tumor size as well as more nuanced imaging features, tumor size was excluded, and no other multicollinearity was found (VIFs < 2). Multivariable Cox analysis identified AFP (hazard ratio (HR), 1.628 [95% CI: 1.113–2.380]; *p* = 0.012) and DLR score (HR, 1.279 [95% CI: 1.051–1.557]; *p* = 0.014) as independent prognostic factors (Table S6). The prognostic models yielded C-indexes of 0.672 (95% CI: 0.621–0.723), 0.679 (95% CI: 0.601–0.757), and 0.642 (95% CI: 0.583–0.701) for RFS in the training, internal, and external test sets.

## Discussion

The VETC, a distinctive histological vascular pattern, has been identified as a predictor of micro-metastasis and poor prognosis in HCC [9]. In this study, we developed a DLR nomogram model for preoperative prediction of VETC and RFS in HCC, which may assist in optimizing treatment decisions and postoperative surveillance. For patients predicted to be VETC+, clinicians might consider postoperative adjuvant strategies such as transarterial chemoembolization (TACE) or sorafenib [10, 11], and adopt intensified radiological surveillance after resection. Individualized RFS prediction generated by the prognostic model may further facilitate personalized follow-up strategies. Nevertheless, the prognostic model demonstrated only modest performance and therefore should be regarded as a complementary rather than a standalone tool for clinical implementation. This limitation may be attributed to the restricted variables included in the Cox regression analysis, given the inherently multifactorial nature of HCC prognosis.

Additionally, the results showed that the DLR nomogram model outperformed the clinical-radiologic model in the external test set while showing comparable performance in the internal test set. It may be due to the limited sample size, leading to minor fluctuations in AUCs, which likely lack clinical significance. Moreover, given its highest F1-scores and comparable AUCs, the main strength of the DLR nomogram lies in its superior classification balance and robustness across datasets, highlighting its practical value rather than clear AUC superiority over the habitat radiomics and DL models. SHAP analysis offers an interpretable DLR nomogram model for clinical application.

Previous studies identified high AFP level, tumor size > 5 cm and intratumor necrosis as independent predictors of VETC, consistent with our clinical-radiologic and DLR nomogram model [9, 28]. In addition, viral hepatitis, absence of capsule and absence of washout were also independent predictors in both models. Viral hepatitis is associated with more aggressive HCC phenotypes [22], while an intact tumor capsule indicates better differentiation and prognosis [29], suggesting associations of VETC with viral hepatitis infection and nonencapsulation. Moreover, angiogenesis activation was a mark of aggressive VETC HCC [30, 31]. Hypoxic conditions emerged as tumor cells proliferated and the distance from the existing vascular network increased [32]. Thus, fibrous stromal hyperplasia caused by neoangiogenesis and hypoxia might indirectly lead to the delayed contrast retention and absence of washout [33].

Conventional radiomics has been widely recognized as an effective tool for VETC prediction by extracting quantitative imaging features from the entire tumor regions [34]. However, it faced limitations in capturing intratumor heterogeneity compared with habitat radiomics [12]. Yang et al [35] integrated traditional imaging and radiomic features with extracellular contrast agents to predict microvascular invasion and VETC. In contrast, our study applied habitat radiomics to delineate homogeneous subregions and better characterize tumor heterogeneity. Using Gd-EOB-DTPA, a hepatobiliary-specific MRI contrast agent, further enabled the assessment of hepatocellular function and HCC differentiation status, which might explain why the optimal habitat radiomics model was developed based on features extracted from HBP. Moreover, the optimal DL model was constructed using CP features, likely because they provided more comprehensive information [36].

Additionally, we developed a Swin Transformer model to predict VETC. Chu et al [37] developed a DL model using 3D convolutional neural networks, which was limited by a relatively small sample size and a lack of a validation cohort. Our study included a larger dataset with an independent external test set, ensuring better reproducibility. Furthermore, the Swin Transformer, as an emerging DL architecture, further improved feature extraction by capturing both global and local information through self-attention mechanisms and window-based attention, achieving flexible and efficient feature extraction [38].

Our study had several limitations. First, the retrospective design may introduce selection bias. Second, the 3D DL architecture is sensitive to data size and quality, highlighting the need for larger multicenter datasets in the future. Third, only MRI images and clinical features were integrated; future studies will incorporate multidisciplinary data, such as digital pathology images, to improve our model. Finally, overall survival analysis was not included; future prospective studies with comprehensive prognostic biomarkers are warranted.

In conclusion, the DLR nomogram model integrating selected clinical-radiologic characteristics with habitat radiomics and DL scores helps predict VETC in HCC and assess the risk for RFS.

## Supplementary information

ELECTRONIC SUPPLEMENTARY MATERIAL

## Data Availability

The data used or analyzed during the current study are available from the corresponding author upon reasonable request.

## Abbreviations

AFP: Alpha-fetoprotein
ALT: Alanine aminotransferase
AP: Arterial phase
APHE: Arterial phase hyperenhancement
AST: Aspartate aminotransferase
AUC: Areas under the curve
CAM: Class activation mapping
CP: Combined phase
DL: Deep learning
DLR: Deep learning radiomics
DSC: Dice similarity coefficient
GGT: γ-glutamyl transferase
HBP: Hepatobiliary phase
HCC: Hepatocellular carcinoma
PP: Portal venous phase
RFS: Recurrence-free survival
ROC: Receiver operating characteristic
SHAP: Shapley additive explanation
VETC: Vessels encapsulating tumor clusters
VIF: Variance inflation factor
VOI: Volume of interest
